# Recognition of Tabes Dorsalis

**Published:** 1901-07

**Authors:** 


### Recognition of Tabes Dorsalis.

Dr. Theodore Diller thinks that too much importance is attached to staggering as a diagnostic symptom of locomotor ataxia, since in a certain per cent. of cases ataxia is never present at any stage, and in the cases in which it is present it does not become manifest until the disease is well advanced.

He says that in almost every instance the chief symptoms which lead the tabesic patient to seek medical advice are (1) lightning pains in the legs; (2) loss of function of the bladder and sexual organs; and (3) double vision or failure of vision.

He concludes: The following symptoms I believe may be said to be the cardinal ones of tabes, and are named in the order of their importance:

1. Failure of knee-jerks.
2. Romberg symptoms (swaying with eyes closed).
3. Argyll Robertson pupil.
4. Lightning pains.
5. Loss of function of the bladder or sexual organs.

With the presence of any three of these symptoms, I believe the diagnosis may with certainty—and with the presence of any two, with probability—be made, when evidence pointing to multiple neuritis, paralytic dementia or cerebro-spinal syphilis is absent.

Among the important secondary signs or symptoms are:

a. Paresthesia, anesthesia or analgesia of the legs.
b. Locomotor ataxia.
c. Paresthesia in the ulna distribution.
d. Optic atrophy.

—*American Medicine,* June 1st.

